# Continuous Storage Root Formation and Bulking in Sweetpotato

**DOI:** 10.12688/gatesopenres.12895.4

**Published:** 2020-07-09

**Authors:** Astere Bararyenya, Phinehas Tukamuhabwa, Paul Gibson, Wolfgang Grüneberg, Reuben Ssali, Jan Low, Thomas Odong, Mildred Ochwo-Ssemakula, Herbert Talwana, Natasha Mwila, Robert Mwanga

**Affiliations:** 1 Department of Agricultural Production, College of Agricultural and Environmental Sciences, Makerere University, Kampala, Central Uganda, Box 7062, Uganda; 2 Crop Improvement, International Potato Center (CIP), Avenida La Molina 1895, Apartado 1558, Lima 12, Peru; 3 Crop Improvement, International Potato Center (CIP), Kampala, Central Uganda, Box 22274, Uganda; 4 Economics, International Potato Center (CIP), Nairobi, Nairobi, ILRI Campus Naivasha Rd, 25171-00603 Lavington, Kenya

**Keywords:** Sweetpotato, yield, continuous storage root formation and bulking, growth pattern, phenotypic variation

## Abstract

This study investigated the phenotypic variation of continuous storage root formation and bulking (CSRFAB) growth patterns underlying the development of sweetpotato genotypes for identification of potential varieties adapted to piecemeal harvesting for small scale farmers. The research was conducted between September 2016 and August 2017 in Uganda. Genotypes from two distinct sweetpotato genepool populations (Population Uganda A and Population Uganda B) comprising 130 genotypes, previously separated using 31 simple sequence repeat (SSR) markers were used. Measurements (4 harvest times with 4 plants each) were repeated on genotypes in a randomized complete block design with 2 replications in 2 locations for 2 seasons. We developed a scoring scale of 1 to 9 and used it to compare growth changes between consecutive harvests. Data analysis was done using residual or restricted maximum likelihood (REML). Data showed a non-linear growth pattern within and between locations, seasons, and genotypes for most traits. Some genotypes displayed early initiation and increase of bulking, while others showed late initiation. Broad sense heritability of CSRFAB was low due to large GxE interactions but higher in other traits  probably due to high genetic influence and the effectiveness of the methodology. A high level of reproducibility (89%) was observed comparing 2016B and 2017A seasons (A and B are first and second season, respectively) at the National Crops Resources Research Institute (NaCRRI), Namulonge, Uganda. Choosing CSRFAB genotypes can more than double the sweetpotato production (average maximum yield of 13.1 t/ha for discontinuous storage root formation and bulking (DSRFAB) versus 28.6 t/ha for CSRFAB, demonstrating the importance of this underresearched component of storage root yield.

## Introduction

Sweetpotato ( *Ipomoea batatas* (L.) Lam, family Convolvulaceae) is one of the most important food crops worldwide, with approximately 106 million tons produced in almost 120 countries from an area of about 8 million ha and an average global yield of 11.1 tons/ha ( FAO, 2016). Asia is the world’s largest sweetpotato producing continent, with 79 million tons, followed by Africa ( FAOstat, 2016). About 75% of this global production is from China alone. A total of 21.3 million tons is produced in Africa, with 48% from the Great Lakes region. In East Africa, the crop is the second most important root crop after cassava and has played an important role as a famine-relief crop during its long history and has recently been reevaluated as a health-promoting food ( Low *et al*., 2017). Uganda ranks as the fourth largest sweetpotato producer in the world after China, Nigeria and Tanzania, with a production of 2.1 million t. In Africa, Uganda is ranked third after Nigeria and Tanzania. Sweetpotato is one of the main staple crops in the food systems of Uganda, Rwanda, and Burundi with a per capita consumption of 50.9, 80.1 and 57.0 kg, respectively ( Table 1).

**Table 1.** Increase in sweetpotato production in East and Central African countries, production data for 1985 and 30 years later for 2015.

Country	Area		Yield		Production		Population		Per annum	
					per year					
	x 1000 ha		tons per ha		x 1000 tons		millions		kg per capita	
	1985	2015	1985	2015	1985	2015	1985	2015	1985	2015
Burundi	87.0	58.6	6.4	9.9	555.0	580.9	4.7	10.2	118.0	57.0
Kenya	50.0	72.2	9.9	17.1	488.0	1232.3	19.7	47.2	24.8	26.1
Rwanda	135.9	139.7	7.1	6.7	979.5	931.0	6.1	11.6	160.1	80.1
Uganda	358.7	454.5	4.6	4.5	1664.0	2045.2	14.7	40.1	113.6	50.9
Tanzania	290.0	746.6	4.6	12.5	303.0	3454.5	21.8	53.9	13.9	64.1
Average	184.3	294.3	6.5	10.1	797.9	1648.8	13.4	32.6	86.1	55.6
**Increase**		**1.60**		**1.06**		**2.07**		**2.44**		**30.45**

Source: FAOstat, 2016

On average and across the 30-year period, the population across East Africa increased by two-and-half-fold while sweetpotato production increased by two-fold. The increase in production was due to a combination of factors varying in different countries but mainly due to increase in area and breeding efforts. This trend resulted in a decrease in per capita production from 86 to 56 kg per person. Statistics in general underestimate production in most annual plants since not all crop production is recorded. Usually, only the main planting season is recorded even though crops are grown over two to three growing seasons per year ( Bararyenya *et al*., 2018a). The most noticeable production increase took place in Tanzania with more than ten-fold increase of tonnage following an almost two-fold increase in growing area and almost four-fold increase in yields. The highest productivity is recorded in Kenya, with more than 17 t/ha/yr, followed by Tanzania (12.5 t/ha/yr). In other East African countries, productivity has more or less stagnated. The yield increase in the region over the 30-year period can be attributed to breeding and release of improved varieties by national breeding programs, and to the introduction of new varieties mainly, by the International Potato Center (CIP). The increase in yield can also be explained by the importance and recent interest in sweetpotato as a food and nutritious crop compared to the early 1990s when it was hardly known.

Despite the giant strides made through breeding and release of 27 high yielding and disease resistant varieties ( Mwanga *et al*., 2016), Uganda has over the last 30 years consistently reported extremely low yields of 4.5 t/ha ( Table 1) compared to the achievable yields of over 40 t/ha under improved conditions. The low yield could be attributed partly to the growing of low yielding and highly disease susceptible landraces by small-scale farmers. Abidin (2004) reported high farmer preference for their landraces, while Sseruwu (2012) associated this preference with lack of farmer desired attributes in the newly released varieties. In Uganda and other East African countries, piecemeal harvesting, characterized by repeated harvesting from the same sweetpotato plants on a mound, ridge or other seedbed over time, is the predominant mode of harvesting among subsistence and commercial sweetpotato farmers. Importantly, this practice is also known in other root and tuber crops including potato, cassava and yam. Recently, Tadesse *et al*. (2017) reported that piecemeal harvesting was the dominant practice among poor potato farmers (60% of respondents) in Ethiopia, whereas the majority of the wealthy and medium-wealthy farmers combined piecemeal harvesting with harvesting all at once. Farmers harvest enough for one or few meal(s), or enough for one market. This is because these crops are very difficult to store, and storage for fresh sweetpotato produce is virtually non-existent. Therefore, the practice allows for in-ground storability and market partitioning during the cropping season. It also creates room for new storage roots to initiate and enlarge for the next harvest. However, breeding and selection have been based on a single harvest and no breeding research has tried to understand the genetics underlying traits associated with these common practices in sweetpotato farming systems. Continuous storage root formation and bulking CSRFAB in sweetpotato is associated with the perennial nature of the crop and allows for longer photosynthetic activity resulting from persistent canopy development leading to increased plant productivity. This is supported in the case of practices in Uganda by the fact that more than 90% (n = 350) of the farmers have no knowledge of the maturity periods of the local sweetpotato varieties they grow ( Bashaasha *et al*., 1995).

Maturity periods in sweetpotato vary with genotype and the environmental conditions under which they are grown. Common external signs of maturity such as senescing and yellowing of leaves do not apply to all varieties. In addition, storage roots do not all mature at the same time, while the storage root formation period is highly variable among genotypes ( Belehu, 2003; Belehu *et al*., 2004; Lowe & Wilson, 1974; Wilson & Lowe, 1973; Yanfu *et al.*, 1989). It is reported that the onset of storage root initiation can occur as early as 7 to 13 days after planting (DAP) ( Du Plooy, 1989), and the total storage root number to form varies from 30 to 112 DAP depending on the genotype and the environmental conditions under which they are grown ( Yanfu *et al*., 1989). Furthermore, sweetpotato storage roots can undergo periods of arrested growth during unfavorable conditions and then continue active growth upon favorable conditions ( Ravi *et al.*, 2009). There are many reported studies on the effects of storage root formation and bulking under controlled conditions on sweetpotato yields ( Gajanayake & Reddy, 2016; Meyers *et al*., 2013; Solis *et al*., 2014; Villordon, 2015) and under field experiments ( Agata, 1982; Belehu *et al*., 2004; Du Plooy, 1989; Enyi, 1977; Gajanayake *et al*., 2014; Lowe & Wilson, 1974; Wilson & Lowe, 1973; Wilson, 1982; Yanfu *et al*., 1989). However, most of the studies were undertaken on single season harvest basis, and no study attempted to understand the CSRFAB patterns overtime under field conditions to focus on improving the trait through breeding.

Knowledge of the growth patterns of storage root formation and bulking traits is critical for crop yield improvement, crop management, and specifically for timing fertilizer application and irrigation. Sweetpotato has been for centuries selected for its starchy roots on seasonal basis and may have been progressively losing its perennial feature. It is well known in other root and tuber crops that yields are a result of the rate and duration of tuberization, which in turn depends on longevity of the leaves, the beginning of storage root formation, and duration of the growth cycle. In almost all experiments, the end time of storage root formation was not defined or properly assessed; number of storage roots was infrequently recorded and maximum number of storage roots was never established. The effect of longer vegetative maintenance periods of green leaves observed in some genotypes has never been investigated in sweetpotato, but it is reported to influence greater productivity in potato ( Çalişkan *et al*., 2007). Despite these deficiencies, the storage root formation and bulking patterns are widely regarded as a key developmental stage in the crop’s life, having profound implications for subsequent growth and development. It was therefore hypothesized that longevity of green foliage due to genetic properties in CSRFAB sweetpotato genotypes is greater than in discontinuous storage root formation and bulking (DSRFAB) genotypes. Consequently, the extended period of green leaves for CSRFAB genotypes will impact storage root formation and bulking leading to a significant increase in yield. This expected variation in yield and yield components is mainly due to longer duration of photosynthetic activity and the great availability of photo-synthesizing material, mostly its green leaves ( Paltridge *et al*., 1984). Thus, the amount of change in the mean value of expected responses associated with a unit increase in growth time, holding all other variables constant, varies with increased growth time periods of some sweetpotato cultivars and produces higher amounts of storage root number and weight. To really understand the evolution of a trait, we need to know whether any variability in that trait can be assigned to genetic effects. If so, and if there is fitness variation associated with the trait; it will be subject to natural selection ( Croston *et al*., 2015). This study investigated genetic variability of CSRFAB and, characterized growth patterns at different development stages to identify possible CSRFAB sweetpotato genotypes in the germplasm collection in Uganda for use as parents in improvement of the trait.

## Methods

### Plant materials and experimental sites

***Plant materials.*** This study utilized 130 genotypes currently used for population improvement in Uganda for various breeding purposes and were screened for CSRFAB traits ( Table 2). The 130 genotypes included two distinct sweetpotato gene pool populations (Uganda A and Uganda B) that were formed to reflect similarity within and divergence between the populations based on 31 simple sequence repeat (SSR) markers ( David *et al*., 2018). The genotypes are maintained by the International Potato Center (CIP) at the National Crops Resources Research Institute (NaCRRI) at Namulonge in Uganda.

**Table 2.** List of genotypes screened for continuous storage root formation and bulking in Uganda, 2016 to 2017.

Code	Name	Code	Name	Code	Name	Code	Name	Code	Name
A1	Carrot C	A27	Apa352	B3	Hma496	B29	Mkn1210	B55	K-566632
A2	Ejumula	A28	Luw1274	B4	Msd380	B30	NASPOT 5	B56	New Kawogo
A3	Mayai	A29	Sponge	B5	Luw1230	B31	Kre723	B57	Bitambi
A4	Naspot5/58	A30	NASPOT 7	B6	Srt43	B32	Ara236	B58	Kbl611
A5	Kbl619	A31	NASPOT 10 O	B7	Srt28	B33	SPK004(CIP)	B59	Mkn171
A6	Kmi61	A32	Kml872	B8	Mle199	B34	Mpg1158	B60	Mle191
A7	Kbl631	A33	Msk1040	B9	Mary	B35	Jonathan	B61	Iga998
A8	Pal133	A34	Dimbuka-Bukulula	B10	Kml942	B36	Kml960	B62	Pal134
A9	Pal94 silk	A35	Oguroilwe	B11	Lir302	B37	Bsh741	B63	Wt-237
A10	Rak786	A36	Kmi159	B12	Kbl650	B38	Bnd145l	B64	Iga994
A11	Mpg1128	A37	Mkn1168	B13	Mpg1151	B39	Srt41	B65	Naveto
A12	Tanzania	A38	Apa335	B14	Mkn1180	B40	Srt01	B66	Xushu18
A13	Kala	A39	Apa323	B15	Luw1257	B41	Wagabolige	B67	Yanshu1
A14	Kbl648	A40	Otada	B16	Kre691	B42	Lir258	B68	Zambezi
A15	Kml956	A41	Ukerewe	B17	Mbr536	B43	Pal148	B69	Jewel
A16	Bsh740	A42	Kmi88	B18	Ksr662	B44	Rak865	B70	Caromex
A17	Silk(1254)	A43	Mpg1148	B19	Mle179	B45	Mpg1122	B71	Resisto cip
A18	Rak819	A44	Iga983	B20	Msd431	B46	Msk1079	B72	Baeuregard
A19	SPK004	A45	Pal108	B21	Mle163	B47	Rak848	B73	Kyabafuruki
A20	Nk259l	A46	Rak835	B22	Magabali	B48	Mugande	B74	Tainung 64
A21	Kml881	A47	Msd384	B23	Kbl618	B49	Ara224	B75	Kre696
A22	NASPOT 9 O	A48	Srt27	B24	Mbr552	B50	Dlp3163	B76	Raihna
A23	Tororo 3	A49	Nk318l	B25	Iga978	B51	Msk1094	B77	Tis9265
A24	NASPOT 1	A50	Mle194	B26	Sowola	B52	Tis9101	B78	199062.1
A25	Rak808	B1	Resisto	B27	Hma490	B53	NASPOT 3	B79	Santa amaro
A26	Mpg1146	B2	Ara209	B28	Apa356	B54	NK1081L	B80	Huarmeyano

***Experimental sites and duration of the study.*** Grüneberg *et al.* (2005) identified Uganda as environmentally diverse and suitable for sweetpotato selection for East African countries, and a breeding platform where crossings and selection in early and later breeding stages were conducted. The results from such environments are extended to all East African countries with one or two more season evaluations for confirmation. Two locations, NaCRRI-Namulonge and National Semi-Arid Resources Research Institute (NaSARRI-Serere) were therefore selected to host the trials for screening potential CSRFAB genotypes adapted to the East African agroecologies. The trials were planted on September 22, 2016 and September 29, 2016, respectively at Namulonge and Serere and harvested from January through April 2017 for the first season (2016B). In the second season (2017A), trials were planted on March 10, 2017 and March 29, 2017, respectively, at Namulonge and Serere and harvested from June to September 2017. The altitude of the sites was around 1,150 meters above sea level with an average day temperature of 22.2°C. Crops were not irrigated and often suffered from low rainfall 4 months after planting. Namulonge is characterized by a tropical rain forest zone with a bimodal rainfall average of 1,270 mm annually and high sweetpotato virus disease (SPVD) pressure, while Serere is in the tall grassland savanna zone with low rainfall and high weevil population ( Table 3).

**Table 3.** Description of study sites used for screening sweetpotato genotypes for continuous storage root formation and bulking in Uganda.

Location	Elevation	Temperature	Rainfall	Soil			
	(masl)	(°C)	(mm)	pH	OM	K	P
					(%)	Cmol/kg	Cmol/kg
NaCRRI (2016B)	1150	24–30	1400–1600	4.8	3.8	0.11	Trace
NaSARRI (2016B)	1140	25–32	1100–1300	4.9	3.7	0.19	Trace
NaCRRI (2017A)	1150	22–29	1550	5.2	3.4	0.76	1.66
NaSARRI (2017A)	1140	23–31	1150–1350	4.9	3.6	0.56	0.92

*Note*. NaCRRI: National Crops Resources Research Institute; 2016B: 2016 second season; NaSARRI: National Semi-Arid Resources Research Institute; 2017A: 2017 first season; masl: meters above sea level; °C: degrees celcius; mm: millimeters; OM: organic matter; K: potassium; P: phosphorus

### Experimental design

Vine cuttings of approximately 30 cm of 3 to 4 nodes each were planted; 20 cuttings of each of the 130 sweetpotato genotypes were planted in a randomized complete block design in 2 locations (Namulonge and Serere) for 2 seasons, 2016B and 2017A (A and B are first and second season, respectively). Each genotype in a plot of five plants had four replications, but for ease of data collection and analysis, the samples were collected as two replications. Plant density was 1 m between rows and 0.3 m between plants within the row. The genotypes were sequentially and destructively harvested at 3, 4, 5 and 6 months after planting (MAP) to allow identification of storage root formation and bulking patterns following the repeated measures design ( Causton, 1994; Hesser, 2015). During each harvest period, four plants were uprooted for above and below ground part data collection. Each row was bordered by two sweetpotato plants at the extreme ends. The plots were kept weed free and no fertilizer or other agro-chemicals were applied.

### Data collection methods

***Growth and development measurements.*** Traits that are known to characterize growth in sweetpotato storage roots were selected. These included: (i) number of harvested plants (NPH), allowing calculation of average values, (ii) total storage root number (SRN), (iii) Total root weight (TRW) measured with a round spring balance scale (Hanson, 8 in x 8 in x 3 in), was used to calculate yield as tons per hectare, (iv) commercial and noncommercial storage root number (CRN & NCRN, respectively) and commercial and noncommercial storage root weights (CRW & NCRW, respectively), allowing the estimation of storage root formation, bulking rate, (v) vine weight (VW), (vi) root system weight (RSW) used to calculate biomass yield (BMY), and (vii) harvest index (HI). Senescence (SEN) was estimated using a scale of 1 to 9, where 1 = no senescence, 9 = severe senescence marked by death/drying ( CIP/AVRDC/IPGR, 1991).

**Figure 1.**
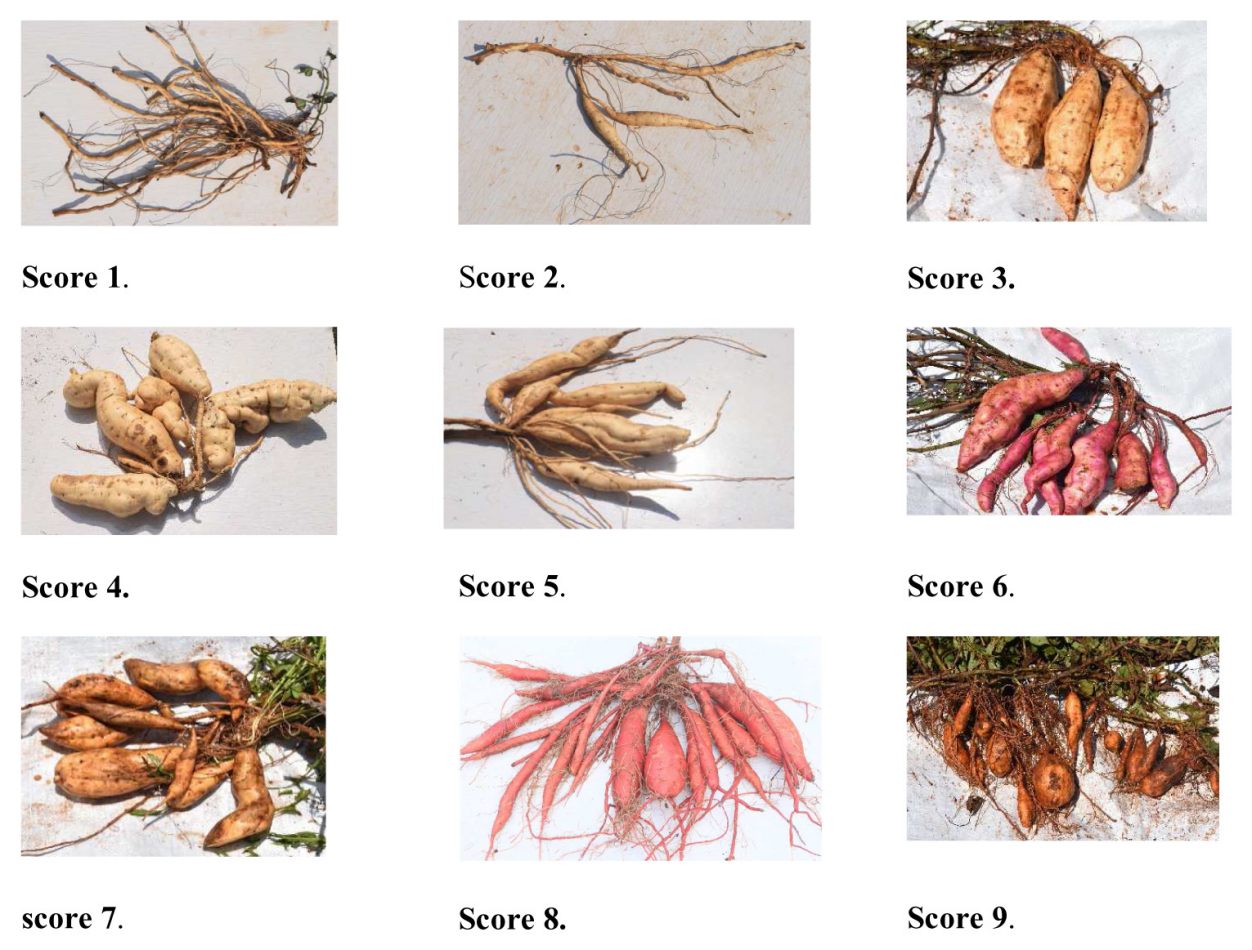
A 1 to 9 scoring scale for continuous storage root formation and bulking in which score: 1 = no visual detectable storage root initiation (SRI) and no visually detectable bulking; 2 = Storage root formation and bulking detectable; 3 = no visually SRI and has unclear levels of bulking; 4 = distinct SRI and 2 clear levels of bulking; 5 = distinct SRI and 3 clear levels of bulking; 6 = distinct SRI and 4 clear levels of bulking; 7 = distinct SRI and 5 clear levels of bulking; 8 = distinct SRI and 6 clear levels of bulking; and 9 = distinct SRI and 7 clear levels of bulking.

***Development of a scale for continuous storage root formation and bulking (CSRFAB).*** To capture the possible overtime initiation and bulking in sweetpotato, we developed a rating scale of 1 to 9. The scale rates the expression of CSRFAB trait for an individual genotype. CSRFAB scores were therefore developed throughout the 2016B growing season. Detailed observation of storage root formation and bulking over 6 months provided comprehensive knowledge of the development of a scale for measuring changes in the CSRFAB trait. The scale was set to measure the changes in root formation and bulking of a given individual genotype. The change in scores would reflect the potential of roots to bulk into storage roots (fleshy or lignified), newly initiated storage roots, their status of bulking and their maturity levels (see Figure 1, scoring scale).

### Data analysis

***Growth model analysis.*** Growth models were estimated using a multilevel, mixed model framework described by Payne (2009) in GenStat 18 ^th^ Edition. Each of the 130 genotypes was maximally compared (4160 times) with the other genotypes in a model with two replications, four harvest times, two seasons and two locations.

To better understand the details in the storage root development patterns across the four harvest times, we analyzed piecewise, storage root growth patterns in different phases of the trial and selected individual plants to represent different growth pattern characteristics. Means were plotted to visualize the growth patterns using Genstat 18 ^th^ edition.

Restricted maximum likelihood (REML) variance components analysis of the phenotypic data was performed using the following general linear mixed model;

$\begin{array}{l} {\text{Y}}_{\text{ijklcqm}}={\text{μ+S}}_{\text{i}}+{\text{L}}_{\text{j}}+{\text{SL}}_{\text{ij}}+{\text{SLR}}_{\text{ijk}}+H_{l}^{1}+H_{q}^{2}+H_{c}^{3}+{\text{SH}}_{\text{il}}+{\text{LH}}_{\text{jl}}+{\text{SLH}}_{\text{il}}+{\text{E}}_{\text{m}}+EH_{lm}^{1}+EH_{qm}^{2}+EH_{cm}^{3}+\\ {\text{SE}}_{\text{im}}+{\text{LE}}_{\text{jm}}+{\text{SLE}}_{\text{ijm}}+{€}_{\text{iml}}+{\text{LEH}}_{\text{jml}}+{\text{SLEH}}_{\text{ijml}}+{\text{ε}}_{\text{ijklcqm}} \end{array}$

Where

Y _ijklm_ is the observed overtime response of genotypes across location and season.

μ, is the overall mean, S _i_ is the effect of the ith season, L _j _is the effect of the jth location, SLR _ijk_ is the effect of the interaction between the ith season in the jth location and the kth replication, $HT_{l}^{1}$ is the effect of linear term l in polynomial model, $HT_{q}^{2}$ is the effect of the quadratic term q in polynomial model, $HT_{c}^{3}$ is the random effect of the cubic term in the polynomial model, SH _il_ is the interaction between season and harvest time, LH _il_ is the random effect of interaction between jth location and h ^th^ harvest time, SLH _iil_ is the effect of the interaction between the ith season and the jth location at the mth harvest time, E _m_ is the effect of the m ^th^ genotype, $EH_{lm}^{1}$ *is the* effect of interaction between mth genotype and lth linear term, $EH_{qm}^{2}$ is the effect of the interaction between ith genotype and qth quadratic term in the polynomial model, $EH_{cm}^{3}$ is the effect of interaction between ith genotype and cth cubic term in the polynomial model, SE _im_ is the effect of interaction between season and genotype, LE _jm _is the effect of interaction between location and genotype, SLE _ijm_ is the random effect of the interaction between season, location and genotype. €SEH _iml_, is the effect of the interaction between season, harvest time and genotypes, LEH _jml_ is the random effect of the interaction between location, harvest time and harvest time, SLEH _ijml_ is the interaction season, location and harvest time, Ɛ _ijklcqm_ is the error associated with all factors involved in the polynomial model.

The F-test denominator for genotypes tested in different locations and seasons ( Bararyenya *et al.*, 2018b) was calculated following Satterthwaite’s formula for the “composite” F-test denominator for genotypes:

Satterthwaite’s formula for the “composite” F-test denominator for genotypes is as shown:

$$\begin{array}{l} Den\;MS=MS\left(GL\right)+MS\left(GS\right)+MS\left(GSL\right)\\ \;\;\;\\ Den\;df=\;\frac{{\left[MS\left(GL\right)\;+\;\;MS\left(GS\right)\;\;+\;\;MS\left(GSL\right)\right]}^{2}}{\frac{MS{\left(GL\right)}^{\text{2}}}{df\left(GL\right)}+\frac{MS{\left(GS\right)}^{2}}{df\left(GS\right)}+\frac{MS{\left(GSL\right)}^{2}}{df\left(GSL\right)}} \end{array}$$

giving Satterthwaite-type approximation of mean squares (MS) and degrees of freedom (df),

where MS = mean square, G = genotype, S = season, L = location, df = degree of freedom, Den df = denominator degrees of freedom ( Satterthwaite, 1946; Snedecor & Cochran, 1967).

## Results and discussion

For growth pattern analysis and phenotypic variability across selected sweetpotato traits, we used a linear mixed model to decompose phenotypic variance (P) into different components: genetic (G) and environmental (E) sources, and their interaction effects (GxE). The interactions between genotype, location and season were significant as were most of the three-way interactions, implying that the growth curve must be optimized for the three factors together, because they modify each other’s effect. Thus, the interaction between season, location and replication (SLR) was highly significant across all the parameters. This is because the replication differences were large in some environments as previously observed by Tumwegamire *et al.* (2016). The variability in replication for an experiment involving clonal crops can easily occur. The main effects for season and location were not significant, as were their interactions. The interaction between season, location and harvest time (SLH) was highly significant (P<0.001) for all the parameters in this study, resulting in non-significance of SL, SH and LH interactions. This implies that harvest time effect differs depending on the level of the location and season on storage root yield (SRY), CSRFAB, storage root diameter (SRD), storage root length (SRL), CRW, CRN, HI and SEN ( Table 4). These results require further investigation to identify the level of influence ( Table 4). For all the traits in this study, the interaction between season, location and genotype (SLE) was highly significant (P<0.001). This indicates that there was wide variability of genotype across locations and seasons and this wide variability can be used for sweetpotato yield improvement in a specific location. However, there is a need to study the stability across locations and seasons for easy selection of the CSRFAB trait.

**Table 4.** Mean squares and F-test of significance for continuous storage root formation and bulking (CSRFAB), storage root yield (SRY) (tons/ha), storage root number (SRNO), vine yield (VY) (tons/ha), storage root diameter (SRD) (mm), storage root length (SRL) (mm), commercial root number (CRN) and weight (CRW), harvest index (HI) and senescence (SEN) of 130 sweetpotato genotypes from Uganda.

SOV	d.f.	CSRFAB	SRY	SRNO	VY	SRD	SRL	CRW	CRN	HI	SEN
S	1	1223.56 ^ns^	2263.89 ^ns^	15284.37 ^ns^	73832.70 ^ns^	2821.05 ^ns^	89273.20 ^ns^	125.34 ^ns^	4381.50 ^ns^	383.10 ^ns^	21.90 ^ns^
L	1	243.40 ^ns^	415.52 ^ns^	1609.44 ^ns^	77317.50 ^ns^	1246.97 ^ns^	16297.00 ^ns^	42.99 ^ns^	0.024 ^ns^	22744.50 ^ns^	7.57 ^ns^
SL	1	0.037 ^ns^	906.99 ^ns^	249.05 ^ns^	28398.30 *	4147.13 ^ns^	9217.60 ^ns^	92.14 *	281.46 ^ns^	1855.40 ^ns^	9.24 ^ns^
SLR	4	13.14 ***	595.92 ***	147.08 ***	3278.10 ***	1021.48 ***	4815.60 ***	7.57 ***	42.20 ***	1235.5 ***	1.65 ***
HLin	1	134.56 ^ns^	5039.56 ^ns^	661.59 ^ns^	3801.60 ^ns^	276.73 ^ns^	2692.20 ^ns^	176.72 *	107.95 ^ns^	22840.60 ^ns^	0.01 ^ns^
HQ	1	0.037 ^ns^	310.48 ^ns^	43.89 ^ns^	1670.50 ^ns^	52.76 ^ns^	34.80 ^ns^	14.44 ^ns^	45.44 ^ns^	60.00 ^ns^	6.35 ^ns^
HC	1	33.212 ^ns^	270.43 ^ns^	35.27 ^ns^	204.70 ^ns^	0.22 ^ns^	1170.50 ^ns^	7.39 ^ns^	4.39 ^ns^	4655.80 ^ns^	8.29 ^ns^
SH	3	21.58 ^ns^	172.93 ^ns^	203.05 ^ns^	13015.70 ^ns^	100.56 ^ns^	1998.90 ^ns^	13.28 ^ns^	11.99 ^ns^	5152.80 ^ns^	6.14 ^ns^
LH	3	61.12 ^ns^	1113.99 ^ns^	1062.79 ^ns^	1937.90 ^ns^	4904.45 ^ns^	34388.40 ^ns^	3.83 ^ns^	847.82 ^ns^	7276.10 ^ns^	3.55 ^ns^
SLH	3	27.55 ***	242.29 **	561.79 ***	1626.00 **	728.56 ***	6705.80 ***	17.01 ***	134.57 ***	111.8 ***	2.90 ***
E	129	12.26 ***	262.85 ^ns^	246.97 ***	1370.60 *	503.38 ***	3801.90 ***	4.23 ***	103.32 ***	2569.60 ***	2.13 ***
HLinE	129	2.73 ***	71.90 ^ns^	39.94 ***	613.30 ***	96.35 *	522.20 **	1.24 *	19.72 ***	269.70 ^ns^	1.13 ^ns^
HQ.E	129	0.99 ^ns^	38.08 ^ns^	11.87 ^ns^	215.40 ^ns^	39.75 ^ns^	141.40 ^ns^	0.81 ^ns^	7.32 ^ns^	154.80 ^ns^	0.46 ^ns^
HC.E	129	1.40 ^ns^	46.53 ^ns^	17.32 ^ns^	329.70 ^ns^	38.37 ^ns^	192.40 ^ns^	0.69 ^ns^	7.78 ^ns^	183.10 ^ns^	0.41 ^ns^
SE	129	4.98 ***	175.22 ^ns^	78.07 ***	1089.30 ^ns^	194.50 **	1451.00 *	1.94 **	36.73 **	1115.90 ***	1.35 *
L.E	128	4.02 *	177.72 ^ns^	81.34 ***	988.80 ^ns^	189.38 **	1263.80 ^ns^	1.95 **	33.26 *	1009.30 ***	0.97 ***
SLE	126	2.90 ***	119.02 ^ns^	45.69 ***	995.00 ***	112.38 ***	1029.10 ***	1.25 ***	24.18 ***	807.70 ***	0.89 ***
SHE	385	1.24 ^ns^	50.31 *	15.50 ^ns^	399.80 ^ns^	54.21 ^ns^	289.10 ^ns^	0.74 ^ns^	7.79 ^ns^	202.40 ^ns^	0.77 ***
LHE	377	1.19 ^ns^	57.48 *	17.24 ^ns^	367.00 ^ns^	72.13 ^ns^	364.60 *	0.93 ^ns^	8.67 ^ns^	254.50 ***	0.50 ^ns^
SLHE	331	1.44 ^ns^	52.67 ^ns^	19.74 *	363.80 ^ns^	59.75 ^ns^	295.50 ^ns^	0.82 ***	7.84 ^ns^	177.40 ^ns^	0.31 ^ns^
Residual	1983	1.36	51.05	16.32	412.50	52.54	339.60	0.45	7.35	193.4 ^ns^	0.46

Note: SOV = source of variation; df: degrees of freedom; CSRFAB = continuous storage root formation and bulking (scored on a scale of 1 to 9, where 1 = no storage initiation and no bulking, and 9 = high storage root initiation & bulking); SRY = storage root yield; SRNO = storage root number per plant; VY = vine yield; SRD = storage root diameter; SRL = storage root length; CRW = commercial storage root weight; CRN = commercial storage root number; HI = harvest index; SEN: senescence (scored on a scale of 1 to 9, where 1 = no senescence, 9 = severe senescence, and death/drying); S = Season; L = location; H = harvest time; E = genotype; HLin: Harvest time linear; HQ = harvest time squared; HC = harvest time cubic (ie. lack-of-fit); SL = season by location interaction; SLR = season by location by replication interaction; SH = season by harvest time interaction; LH = location by harvest time interaction; SLH = season by location by harvest time interaction; SE = season by genotype interaction; L.E = location by genotype interaction; SLE = season by location by genotype interaction; SHE = season by harvest time by genotype interaction; LHE = location by harvest time by genotype interaction; SLHE = season by location by harvest time by genotype; * = significant at 0.05; ** = significant at 0.01; *** = significant at 0.001; ^ns^ = non-significant.

The derived overall means ( Table 5) suggest the existence of determinate and extended growth maturity stages among genotypes. High variation (P<0.001) of storage root formation (root initiation) and bulking was observed using the 1 to 9 scoring scale for CSRFAB. This suggests that the scale can be used to differentiate and evaluate the CSRFAB trait among sweetpotato genotypes.

**Table 5.** Variance components, within and across environment heritability estimation for nine characters associated with continuous storage root formation and bulking in sweetpotato.

Trait	σ ^2^_R_	σ ^2^_SLG_	σ ^2^_LG_	σ ^2^_SG_	σ ^2^_G_	*H1*	*H2*	H(%)
CSRFAB	1.4	0.2	0.1	0.1	0.2	68.2	53.7	50.5
SRY	51.1	8.8	3.8	3.7	0.9	77.2	30.9	11.1
SRNO	16.3	3.9	2.4	2.2	7.7	74.9	53.2	67.2
VY	412.5	8.8	3.8	3.7	4.7	68.2	24.9	19.9
SRD	52.5	9.2	4	3.8	4.9	67.2	63.1	38.3
SRL	339.6	9.2	4	3.8	4.9	73.5	60.8	22.5
CRW	0.4	9.4	4.1	3.9	5	69.9	69.4	44
CRN	7.4	9.4	4.1	3.9	5	73.1	56.8	43.2
SEN	0.5	9	3.9	3.7	4.8	46.7	58.0	44

Note. H: broad sense heritability across location and season; H1: within environment broad sense heritability across 2 seasons for NaCRRI; H2: within environment broad sense heritability across 2 seasons for NaSARRI; **σ ^2^_R_**: residual variance; σ ^2^_G_: genotypic variance; σ ^2^p: phenotypic variance; **σ ^2^_SLG_**: variance due to season by location by genotype interaction; σ ^2^_LG_: variance due to location by genotype interaction; **σ ^2^**_SG_: variance due to season by genotype interaction; **σ ^2^**_GxE_: genetic by environmental variance; SRNO: storage root number per plant; SRY: storage root yield; VY: vine yield; SRDIA: storage root diameter; SRLG: storage root length; CRW: commercial root weight: CRN: commercial root number; VW; vine weight; CSRFAB: continuous storage root formation and bulking; SEN: senescence

### Variance component analysis and heritability estimates of selected sweetpotato growth traits

***Variance component analysis and heritability estimates of selected sweetpotato growth traits in a nonlinear model structure.*** The genotypic variance among the nine traits associated with CSRFAB varied from 0.9 (CRW) to 7.7 (SRNO). Genotypic variance was high for SRNO (7.7), CRN (5.0) and CRW (5.0) ( Table 5). The phenotypic variance was high for VY (449.8), SRL (378.5) and SRY (84.6) and varied from 2.4 (CSRFAB) to 449.8 (VY). The residual variance was extremely high for VY (412.5) and SRL (339.6). These two particular traits are influenced by continuous growth. While some genotypes continue to increase in biomass weight, others die by senescence, likewise for vine length. The error and the GxE variances for CSRFAB are not big compared to its genetic variance. This implies that the scale used to measure the trait is precise and the trait is not influenced much by the environment. Heritability was relatively high for SRNO (67.2%) and CSRFAB (50.5%). The low residual variance and high heritability observed for CSRFAB confirmed the precision of the method used to study the CSRFAB trait in sweetpotato.

### Genetic effects on yield of CSRFAB genotypes in sweetpotato

We performed a harvest basis analysis and calculated the corresponding heritability for each trait to understand the dynamics of breeding values across harvest times. We targeted the traits that can affect the final yield. Thus, the genotypic variances among seven main traits associated with CSRFAB varied from 0 to 72.46 ( Table 6). Genotypic variance was high for HI at 120 DAP (72.46) and VY at 180 DAP (59.91) ( Table 6). Zero variance was recorded for VY (120 DAP), HI (150 DAP), SEN (180 DAP) and weevils (180 DAP). The zero (or negative) variance components could be due to an artifact of the optimization algorithm that includes a non-negative constraint; a negative variance component could also represent competition effects between adjacent plots. These results imply that there is no significant change in VY, HI, SEN and weevil damage within the population for the respective growth periods. Since heritability is influenced by genetic variance, it was directly affected at the same traits and harvest periods, (ranging from 0 to 67%). The 0% heritability implies that the effect of the traits is moving to fixation, that is, its frequency in the population is very high. This is because polyploidy crops are dominated by large mutations with small effects and the genetic fixation pattern is affected by interactions between cosegregating alleles ( Kopp & Hermisson, 2009). If we score the effect of the allele(s) regulating the trait having no heritability in the population, the result would mean in genetic terms, that the allele frequency in the population is 100%, therefore the genetic variance at the loci of these genes is zero, so any variance in the corresponding phenotypic traits cannot be attributed to the non-existent genetic variance. These results need further investigation especially in the case of weevil resistance mechanisms as no significant increase in weevil infestation was observed in late harvest ( Bararyenya *et al.*, 2018b). The rate in storage root bulking resulting in the available high green biomass may compete with weevil infestation. Broad sense heritability was relatively high for CSFRAB (50.5%) compared to other yield component traits indicating a better yield prediction using the scale.

**Table 6.** Estimates of genotypic (σ ^2^g) variance and broad sense heritability (H) within harvest time across environments for seven traits associated with continuous storage root formation and bulking.

	Genetic variance (σ ^2^g)					Broad sense heritability H (%)				
Trait	90DAP	120DAP	150DAP	180DAP	Comb.	90DAP	120DAP	150DAP	180DAP	Comb.
SRNO	3.29	10.08	6.46	8.06	8.84	47	67	62	58	70
SRY	2.84	2.32	4.04	0.81	2.43	26	20	23	4	24
VY	14.53	0.00	4.57	59.91	26.50	19	00	6	35	46
HI	29.80	72.46	0.00	36.25	75.26	43	61	0	29	61
SEN	0.01	0.02	0.07	0.00	0.07	12	19	22	0	56
WEEVIL	0.02	0.00	0.24	0.00	0.08	31	0	13	0	45
CSRFAB	0.23	0.51	0.10	0.43	0.45	35	62	49	55	69

Note. **σ ^2^g**: Genetic variance; DAP: days after planting; Comb.: combined estimate; SRNO: storage root number; SRY: storage root yield; VY: vine yield; HI: harvest index; CSRFAB: continuous storage root formation and bulking; SEN: senescence.

We found temporal dynamics of genotypic influence on overall trait development ( Table 6). In the early growth phase, genotypic variance was mostly low. As plants grew, genotypic factors became in general more important. The increasing genetic effect was observed up to about 120 DAP and decreased thereafter. After 120 DAP, the genetic effect became relatively less important. This can be partly explained by the drought stress which became more important 4 months after planting (MAP). Although less obvious, the opposite pattern was seen in the growth recovery phase (after 5 MAP), likely due to growth resumption resulting in the decline in overall phenotypic differences between CSRFAB and DCSRFAB plants. The investigated highly correlated traits with CSRFAB showed dynamic changes in heritability during the entire plant growth stage ( Table 6). SRNO and CSRFAB showed similar patterns of heritability over time. We found that heritability of SRNO and CSRFAB increased in early growth stages and then decreased during drought stress, occurring between two consecutive rainy seasons and then increased thereafter during the growth recovery period occurring with the onset of the rainy season. These results are supported by Tuberosa (2012); quantitative traits reflecting the performance of crops under drought conditions tend to have low to modest heritability ( Table 6).

### Identification of storage formation and bulking growth patterns and their characterization

**Figure 2.**
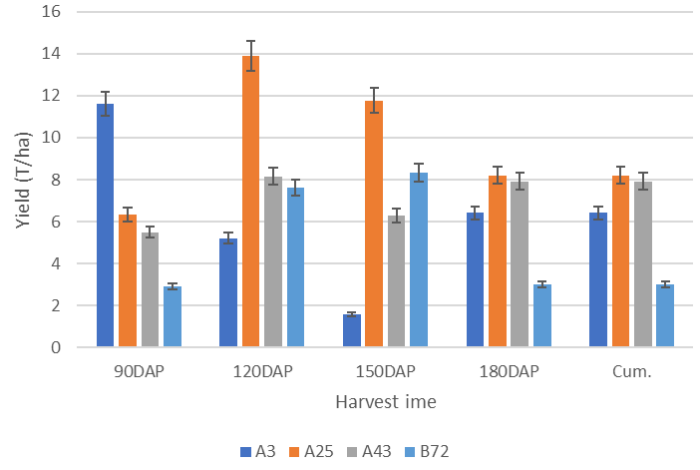
Growth pattern for discontinuous storage root formation and bulking (DCSRFAB) of four sweetpotato genotypes representing different growth patterns over four harvest times (averaged across four environments). Growth trend for genotypes A3, A25, A43 and B72 showing a fully DCSRFAB trait which generally increases the yield up to 120 days after planting (DAP) and then decreases overtime. The cumulative yield is generally lower than the maximum yield due to the yield decreasing in late stages. Note: DAP = days after planting, Cum. = cumulative yield.

**Figure 3.**
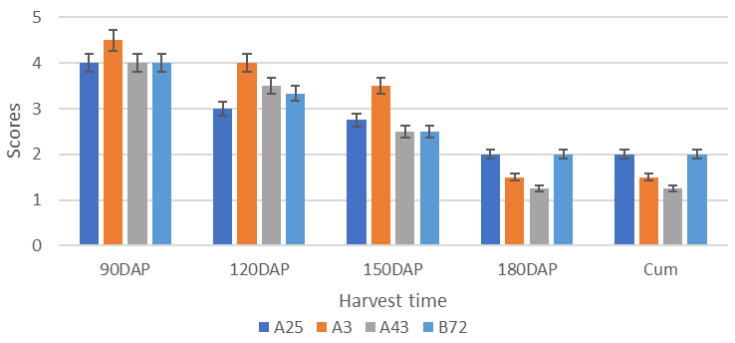
Growth trend of scores over four harvest times for discontinuous storage root formation and bulking genotypes A3, A25, A43 and B72 decreases due to progressive reduction of newly formed storage roots and similar bulking across all storage roots of the same genotype. Note: DAP = days after planting; CSRFAB = Continuous storage root formation and bulking; Cum. = Cumulative scores. DCSRFAB = discontinuous storage root formation and harvesting. Trend of scores over four harvest times for DCSRFAB genotypes A3, A25, A43 and B72 decreases due to progressive reduction of newly formed storage roots and similar bulking across all storage roots of the same genotype. The cumulative scores are generally lower than the respective maximum scores due to to the rate of CSRFAB decreasing in late stages.

**Figure 4.**
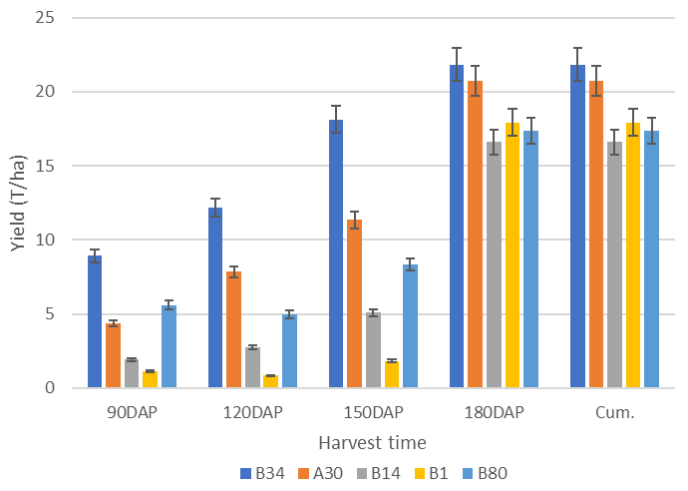
Growth pattern of continuous storage root formation and bulking of four sweetpotato genotypes representing different growth patterns over four harvest times (averaged across four environments). Note: DAP = days after planting; Cum. = Cumulative yield. Growth trend for genotypes A30, A14, B1 and B80 shows CSRFAB with increased yield overtime. The yield increase starts slowly and drastically increases at a late stage (150 DAP). The cumulative yield is the same with the last harvest (180 DAP).

**Figure 5.**
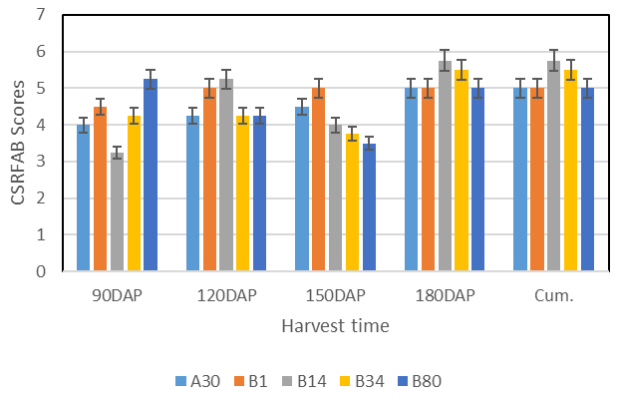
Growth pattern for continuous storage root formation and bulking (CSRFAB) of four sweetpotato genotypes representing different growth patterns over four harvest times (averaged across four environments). Note: DAP = days after planting; CSRFAB = Continuous storage root formation and bulking; Cum. = Cumulative scores. Scores show a trend which is similar across the four harvest times suggesting a possibility of predicting CSRFAB genotypes from early stages upwards. Genotypes A30, B1, B14 and B80 exhibit continuous bulking over the four harvest times. Cumulative scores were the same with 180DAP confirming the precision of the scale.

We have proposed in this study a method that can be used to estimate changes ( Figure 2 to Figure 5) in the CSRFAB trait and therefore estimate the potential maximum yield of a sweetpotato genotype. The scoring method developed in this study effectively measured changes in the CSRFAB trait. This is supported by its low residual variance (1.4) compared to SRN (16.3) and SRY (51.1) ( Table 4) and its high broad sense heritability of 50.5% compared with heritability of SRN (67.2%) and yield (11.1%) ( Table 5). Two types of growth patterns were identified according to the lifetime of sweetpotato genotypes. The first type which has determinate storage root formation and bulking is characterized by a rapid and short vegetative growth period, followed by a senescence period until the leaves die off. This period is also characterized by a quick maximum yield obtained about 90 to 120 DAP ( Figure 2) for most DCSRFAB genotypes. Respective CSRFAB scores for the same genotypes declined overtime due to lack of new root formation and similar level of bulking for mature storage roots ( Figure 3). Changes between successive harvest times were generally negative for DSRFAB genotypes. The cumulative yield changes, as were the cumulative score changes of DCSRFAB genotypes were therefore lower than the respective maximum single harvest yields and the rate of CSRFAB decreases in late stages. The second type of growth is characterized by a prolonged vegetative growth and a late switch-over time to reproductive phase ( Figure 3 and Figure 5). The cumulative yield changes over time, as were the cumulative score changes, of CSRFAB genotypes displayed similar results with the last harvest (180 DAP) confirming the accuracy of the scale to predict yield changes over time. The change responses were positive for CSRFAB genotypes and the cumulative responses increased overtime ( Figure 4) for yield and the increase was not significant ( Figure 5) for the CSRFAB scores suggesting a possibility of predicting CSRFAB genotypes responses from early growth stages. During this period the yield increase starts later and increases drastically after 150 DAP ( Figure 4) for most CSRFAB genotypes. Respective CSRFAB scores of the same genotypes did not change overtime due to continuous root formation and bulking ( Figure 5) suggesting the ability of the scoring scale to predict CSRFAB genotypes at early growth stages. This period increased drastically yields of CSRFAB genotypes, however, the maximum yields were not observed in this trial due to continued vegetative growth. According to Paltridge & Denholm (1974) the variation in yield observed may be due to the variation in their actual rates of dry matter production and the individual switchover time of the genotype to raise its maximum yield, referred to as the optimum growth pattern. The optimum growth pattern for determinate sweetpotato genotypes is of a two-phase plant growth, with the switchover occurring at the instant the plant becomes limited by restriction of its access to light (senescence). Subsequent values of the growing cycle which give maximum yield per unit time of harvest occur after these periods for indeterminate growth which are functions of above ground vegetative (leaves) lifetime. Each successive maximum is larger than the last, so one might expect the plants to evolve longer lifetimes and correspondingly longer periods of indeterminate (CSRFAB) growth. The intensity of senescence strongly varied (P<0.001) among genotypes and was negatively correlated with CSRFAB ( Table 7). The yield rate of change between the 120 DAP and the 150 DAP was always negative. For the CSRFAB ( Figure 3 & Figure 5), all the mean predictions and changes were positive. Yield increased up to more than 20-fold and the increase was genotype dependent.

**Table 7.** Yield means of 10 most distinct discontinuous (DSRFAB) and continuous (CSRFAB) genotypes at different harvest times showing overall best yielding genotypes in combined environments.

DSRFAB genotypes					CSRFAB genotypes				
Genotype	90DAP	120DAP	150DAP	180DAP	Genotype	90DAP	120DAP	150DAP	180DAP
MPG1146	8.23	10.49	17.46	13.17	SPK004	6.47	4.43	10.34	28.64
Ndimbuka	7.18	3.47	6.29	5.83	Kala	4.18	6.72	7.41	24.26
NASPOT 1	6.56	3.19	7.63	3.99	BSH740	5.31	5.67	8.15	18.62
Otada	6.49	3.56	9.03	4.08	KML872	4.31	6.59	12.16	21.44
MPG1128	6.11	6.41	17.33	5.29	NASPOT 9 O	3.52	8.83	4.17	10.37
MSK1040	5.97	9.12	11.97	11.83	Mayai	9.69	11.6	3.63	9.72
RAK786	5.13	2.8	6.36	2.96	Ukerewe	5.47	5.1	5.5	11.59
KMI88	5.03	6.4	7.91	8.52	NASPOT 7	2.68	6.98	7.89	13.88
KBL648	4.6	9.54	8.95	9.33	APA352	1.12	2.95	2.95	8.87
Jonathan	4.6	7.62	17.36	9.54	RAK819	2.92	8.3	5.19	11
**Mean**	6.29	4.48	7.35	4.87	**Mean**	3.54	5.95	7.09	9.83
**Max**	16.71	12.98	17.6	13.17	**Max**	9.82	15.45	19.57	28.64
**Min**	1.24	0.1	0.97	0.3	**Min**	0.21	0.65	0.22	2.41
**SED**	7.67	6.4	7.43	8.55	**SED**	7.67	6.4	7.43	8.55

DAP: Days after planting; DSRFAB: discontinuous storage root formation and bulking; CSRFAB: continuous storage formation and bulking; Comb.: combined; Max: maximum; Min: minimum; SED: standard error difference.

These results are in agreement with the basic growth curves in many crop plants ( Schurr *et al.*, 2006; Yanfu *et al*., 1989). The rapid growth in the first stage for determinate or DSRFAB genotypes could be attributed to early maturing genotype properties in which there is more energy invested in biomass production for early remobilization for storage root bulking and this plant growth expression is also reported in other annual crops ( Wenk & Falster, 2015). However, the maximum yield in this period was low and was limited by the available amount of green biomass which affected the bulking rate. Paltridge *et al.* (1984) reported similar common characteristics in which many plants have a rather sharp transition between the vegetative and the reproductive stages of growth. If the time of switch over is small, the amount of green leaf would be small and the subsequent rate of production and final yield would be also low. In this phase, the time of switchover was before 90 DAP. Sweetpotato continues to grow and branch if environmental conditions are favorable, due to its perennial habit, but the leaves formed earlier in the growing season start to fall and the total number of leaves and leaf area decrease toward the end of the growing season ( Somda & Kays, 1990). Determinate or DSRFAB genotypes lost their ability to initiate new shoots at around 120 DAP and the VW decreased drastically. These results agree with findings of Paltridge & Denholm (1974) and Bhagsari (1990) in which most annuals exhibit a single reproductive phase, often with a sudden onset.

The senescence phenotype was less observed in indeterminate types, although greenness intensity was reduced at 120 DAP which resumed and drastically increased (high positive quadratic slope) from 150 DAP when rains came back. Bhagsari (1990) reported similar results that sweetpotato cultivars maintained leaf area index to intercept a major portion of sunlight until harvest, and leaf area growth significantly differed depending on cultivar.

The slow increase in most of the yield component parameters in CSRFAB genotypes resulted in low storage root yield in the first growth phases ( Figure 3 & Figure 5). This can be explained by the fact that indeterminate genotypes invest resources for maintenance at earlier stages including root elongation for nutrition purposes and biomass production to maintain vegetative growth. This resulted in strong vegetative biomass increase for CSRFAB genotypes. For these genotypes the time of switchover to reproduction was very late (around 150 DAP). This was the last growth phase where yield increased drastically in CSRFAB corresponding to the switch to reproduction (onset of flower and seed) and storage root remobilization in sweetpotato in which the plant invests its vegetative resources in increasing the sink capacity. Because of high availability of resource/energy in upper biomass, sink strength is increased leading to increased productivity.

For sweetpotato breeders and practitioners, harvest index (HI) is difficult to estimate due to the problem of measuring its components. For instance, the time of harvest influences greatly the value of HI because storage root bulking is likely to vary progressively from the onset of storage bulking and increases with the biomass translocation into storage roots. However, the senescence reduces the above-ground biomass progressively leading to high and unrealistic values of harvesting index. Therefore, HI of sweetpotato varies greatly with the time of harvest ( Bararyenya *et al.*, 2018b). This variation is also influenced by other environmental factors including wet or dry conditions during harvest which increase or decrease weight due to high or less moisture. Similar results in other root and tuber crops are reviewed by Hay (1995).

Overall, the starting yield was low at 90 DAP (overall mean) and increased progressively as growth time increased ( Table 7). This relationship is well explained by the variation in the data (R ^2^ varied from 38% to 65%). High yielding genotypes showed consistently maximum yields in the population ( Bararyenya *et al.*, 2018b) while low yielding genotypes showed inconsistent relationship. The maximum yield recorded across locations and seasons was 16.7 t/ha and 28.6 t/ha at the first harvest and the last harvest, respectively ( Table 7). This implies that the frequency of genotypes that increase yield overtime is high. In this trial, some genotypes reached maximum growth earlier, others late, leading to high variability in growth related parameters.

On average, there was a two-fold increase in yield from the 90 DAP to 180 DAP. The 10 most distinct DSRFAB genotypes were, MPG1146 (A26), Dimbuka-Bukulula (A34), NASPOT 1 (A24), Otada (A40), MPG1128 (A11), MSK1040 (A33), RAK786 (A10), KMI88 (A42), KBL648 (A14) and the 10 most distinct CSRFAB were, SPK004 (A19), Kala (A13), BSH740 (A16), KML872(A32), NASPOT 10 O (A22), Mayai (A3), Ukerewe (A41), NASPOT 7 (A30), APA352 (A27) and RAK819 (A18). These contrasting genotypes for CSRFAB can be used in breeding programs to study and improve sweetpotato yield in CSRFAB genotypes. The average yield at two locations in two seasons shows that you can more than double sweetpotato production by choosing high yielding genotypes ( Table 7 with maximum yield of 13.17 t/ha for DSRFAB versus maximum of 28.64 t/ha for CSRFAB). Similar results have been reported ( Çalişkan *et al*., 2007) in which there were significant differences among the locations, 60 to 70 t/ha of storage root yield being obtained by choosing high-yielding varieties.

### Variability and distribution of 130 genotypes over 4 harvest times

**Figure 6.**
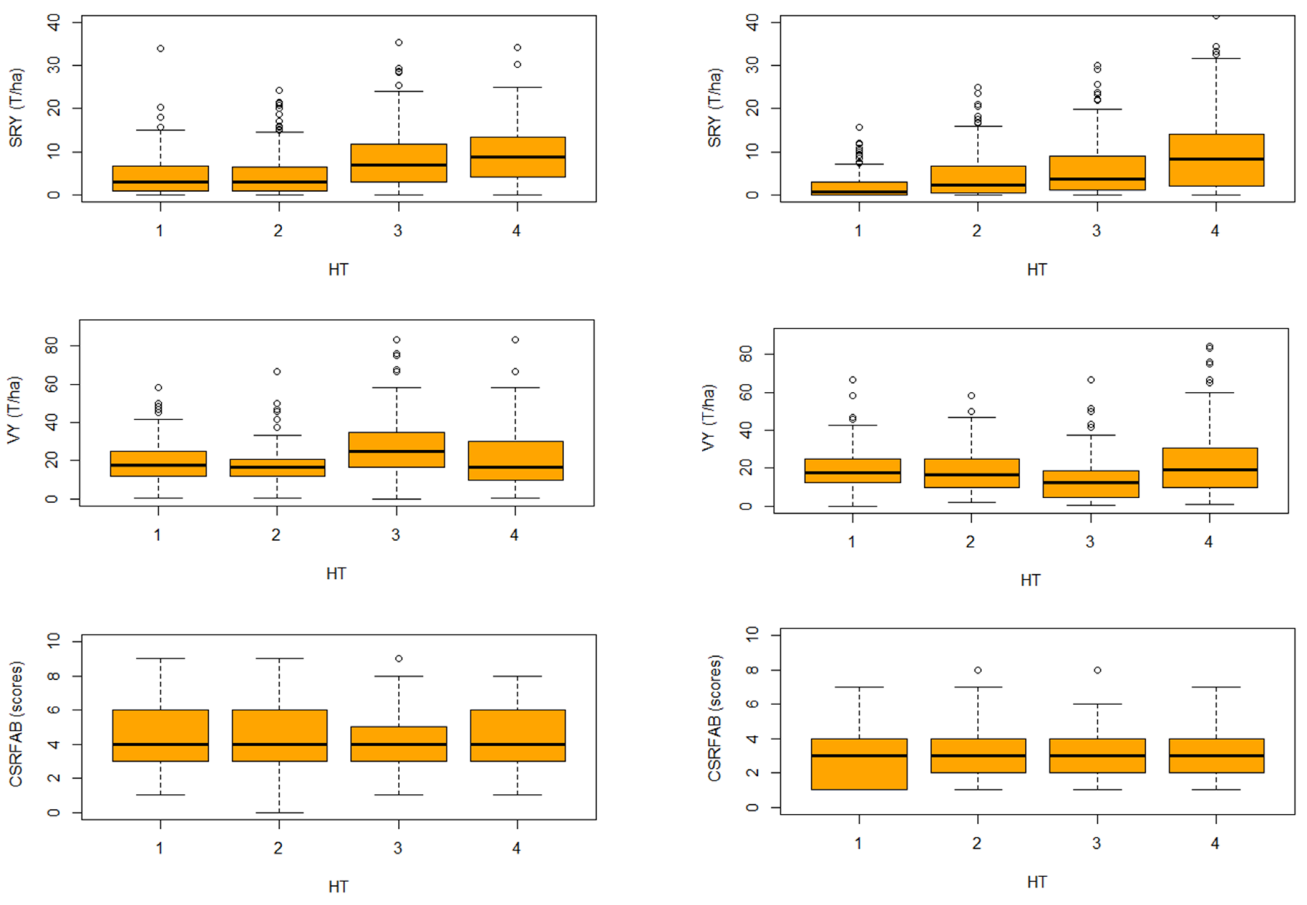
Two seasons (2016B & 2017A) boxplot comparison showing overall variability and dispersion of storage root yield (SRY), vine yield (VY) and continuous storage root formation and bulking (CSRFAB) over 4 harvesting times (HT) among 130 sweetpotato genotype at the National Crops Resources Research Institute (NaCRRI), Namulonge. Note: SRY = storage root yield; VY = vine yield; HT = harvesting time; 1 = 90DAP; 2 = 120 DAP; 3 = 150 DAP; 4 = 180 DAP.

**Figure 7.**
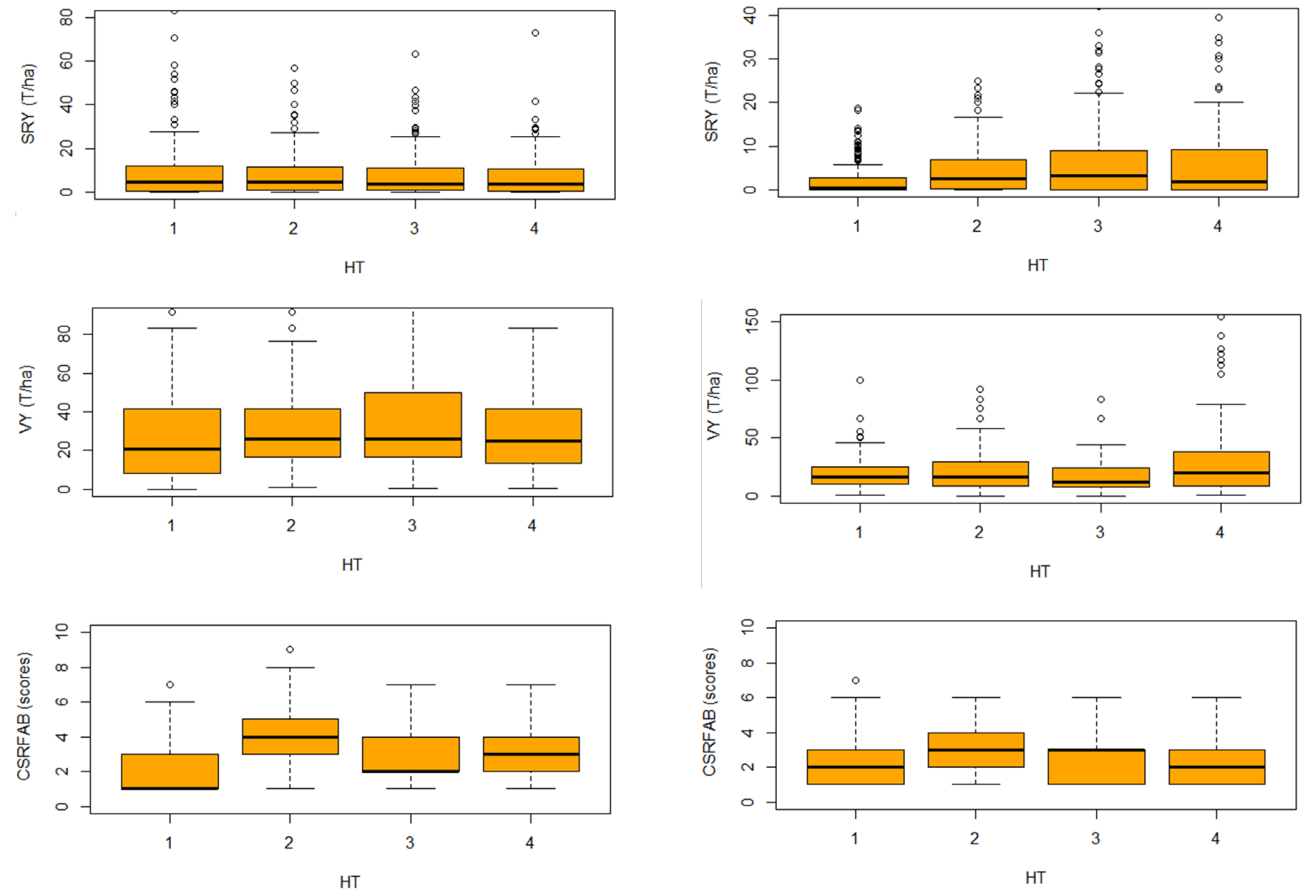
Two seasons (2016B & 2017A) boxplot comparison showing overall variability and dispersion of storage root yield (SRY), vine yield (VY) and continuous storage root formation and bulking (CSRFAB) over 4 harvest times (HT) among 130 sweetpotato genotypes at the National Semi-Arid Resources Research Institute (NaSARRI), Serere. Note: SRY = storage root yield; VY = vine yield; HT = harvesting time; 1 = 90DAP; 2 = 120 DAP; 3 = 150 DAP; 4 = 180 DAP.

For SRY and VY the first two box plots are comparatively equally shorter than the third and fourth box plots ( Figure 6). This suggests that, overall, genotypes have little variation over the first two harvest times. It also suggests low variation between time of harvest during this phase. The third and fourth box plots are higher than the first two box plots for SRY and VY. This suggests that genotypes have quite different growth patterns from the two first harvest times and the two last ones. It also suggests high variation among the genotypes. The last two box plots in each sub-figure show obvious differences within each box plot and the two first box plots. This suggests an area of difference that could be explored further. The four sections of the box plots are uneven in size. This shows that many genotypes have a similar growth pattern during the early growth phase, but in a later phase genotypes are more variable in their growth pattern. The long upper whisker shows that genotype growth varied amongst the most positive quartile group, and very similar for the least positive quartile group. This suggests a need for further exploration. The medians of the two first harvest time plots (which generally will be close to the average) are at the same level. However, the box plot of the last two harvest times shows very different distribution means. Variation and distribution of CSRFAB scores across location and seasons consistently remained almost the same ( Figure 6 and Figure 7), suggesting an effective response prediction of the scores from the beginning.

### Relationship between associated growth traits and CSRFAB in sweetpotato

**Figure 8.**
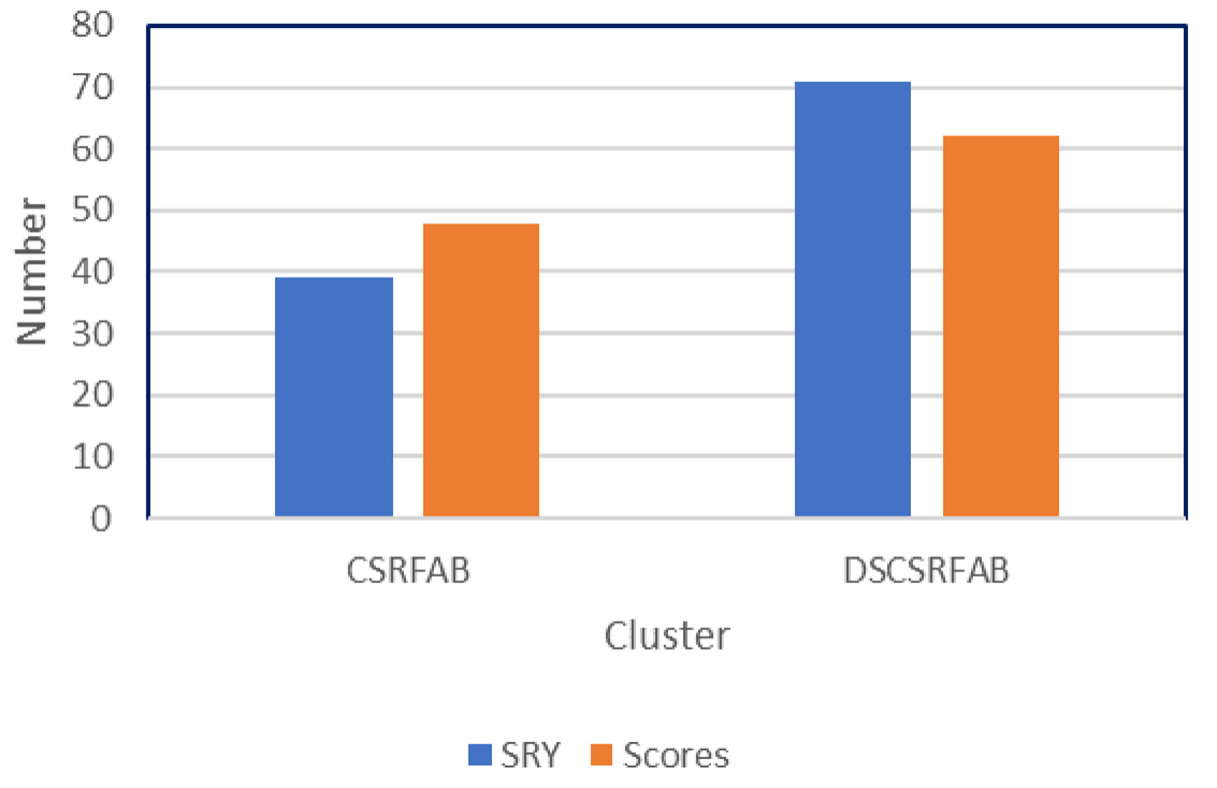
Comparative accuracy of screening continuous growth using yield increase overtime evaluation and using a 1 to 9 scale (discontinuous storage root formation and bulking (DSRFAB) and continuous storage root formation and bulking (CSRFAB) genotypes. Note. SRY: storage root yield.

CSRFAB scores were highly and positively correlated with most of the yield component traits ( Table 8). This implies that CSRFAB is also a component of yield. In other words, the higher the CSRFAB scores of a sweetpotato genotype, the higher the yield of the genotype. CSRFAB scores were negatively correlated with SEN which confirms our hypothesis in which a CSRFAB genotype should maintain vegetative growth to continuously provide source/inputs for sink storage roots. The negative correlation with VW needs further investigation, however, the competition of source and sink activities in the plant, effects of pests and diseases such as nematodes and Alternaria stem blight ( *Alternaria bataticola*) and SPVD could be among the causal factors. It is possible to cease vegetative growth and continue survival for an extended period, but most varieties reduce their vegetative weight following the storage root bulking peak ( Venus & Causton, 1979). The comparison of a classic method of evaluating growth change (analyzing yield mean change between two consecutive harvests) and the developed scale for measuring CSRFAB traits produced consistent results with CSRFAB scores. This is supported by high heritability and low residual variance observed for CSRFAB versus yield and other component parameters. Using the developed scale, 48 genotypes were clustered among CSRFAB genotypes, whereas 62 genotypes clustered among DSRFAB ( Figure 5). These results were reproduced in the following season, and 41% were common to the two methods in 2016B versus 46% in 2017A ( Figure 8).

**Table 8.** Pearson’s correlation coefficients of CRN, CRW, CSRFAB, HI, SEN, SRD, SRL, SRY, VW and VY for 130 sweetpotato genotypes across two locations (Namulonge and Serere) and two seasons (2016B and 2017A) (N = 4160).

	CRN	CRW	CSRFAB	SEN	SRD	SRL	SRNO	SRY	VY
CRN	-								
CRW	0.84***	-							
CSRFAB	0.67***	0.46***	-						
SEN	0.08	-0.05	0.01	-					
SRD	0.74***	0.70***	0.63***	-0.12	-				
SRL	0.75***	0.68***	0.71***	-0.12	0.86***	-			
SRNO	0.90***	0.65***	0.82***	0.09	0.68***	0.72***	-		
SRY	0.74***	0.74***	0.53***	-0.08	0.80***	0.72***	0.70***	-	
VY	-0.29***	-0.25***	-0.16*	-0.21*	-0.17*	-0.18**	-0.25**	-0.14	-

Note. CRN: commercial storage root number; CRW = commercial storage root weight; CSRFAB = continuous storage root formation and bulking (scored 1–9); HI = harvesting index; SEN = senescence (scored 1 to 9); SRD: storage root diameter; SRL: storage root length; SRN storage root number; SRY = storage root yield; VW = vine weight; VY = vine yield.

### Accuracy of CSRFAB scoring method

We characterized in this study growth patterns associated with CSRFAB in sweetpotato and compared the accuracy of CSRFAB scores and the classic method used to measure growth change overtime (compared yield change overtime) ( Figure 8). It was hypothesized that CSRFAB genotypes potentially increase yield over time, therefore, the trait can be screened for by measuring yield change overtime. This method, holding other factors constant, shall produce the same results of scoring for the trait using the developed 1 to 9 scale. We investigated this relationship in our data. Overall, the yield change analysis over four harvest times grouped the 110 genotypes (without missing data) into 39 CSRFAB and 71 DSRFAB genotypes. Using the developed scale, 48 genotypes clustered among the CSRFAB genotypes, whereas 62 genotypes clustered together as DSRFAB ( Figure 8).

## Conclusion

This study highlights three important results: 1) the CSRFAB trait can be exploited to provide additional yield in sweetpotato 2) the 1 to 9 scale developed provided consistent scores across replications, and reflected well the growth patterns observable phenotypically, 3) the method of analyzing growth variables over harvest times revealed distinct growth patterns among genotypes. These patterns identify which sweetpotato genotypes are likely to be suited to piecemeal harvesting. The methodology introduced here is expected to be useful in other root crops as well.

This study showed that CSRFAB genotypes differentially increased yields up to 779% and discontinuous genotypes reduced yield after crop maturity up to 85% for determinate genotypes. Five months after planting (150 DAP) is proposed as the ideal scoring time for this scale, however, there is need for further work in different agroecologies to validate the reliability of the results. The sweetpotato genotypes used in this study are highly variable for CSRFAB and breeding to improve the trait should be feasible due to its high heritability. Genotypes most distinct for CSRFAB were, SPK004, Kala, BSH740, KML872, NASPOT 9 O, Mayai, Ukerewe, NASPOT 7, APA352 and RAK819, and those distinct for DCSRFAB were, MPG1146, Dimbuka-Bukulula, NASPOT 1, Otada, MPG1128, MSK1040, RAK786, KMI88, and KBL648. The highest CSRFAB yielder, SPK004 (28.6 t/ha) outperformed by 15.4 t/ha (117%) the highest DCSRFAB yielder, MPG1146 (13.2 t/ha) across locations and seasons. Delaying in harvest leads to final yield losses for DCSRFAB while it enhances final yield of CSRFAB genotypes. These genotypes can be used in conventional sweetpotato breeding for yield improvement. For sweetpotato breeding, the CSRFAB genotypes are recommended for the improvement of sweetpotato varieties suitable for piecemeal harvesting in small scale farming systems, while the discontinuous genotypes are recommended for the development of early maturing sweetpotato varieties. However, the trait needs much more understanding of the physiology of sweetpotato. Therefore, linking these phenotypic patterns with causal genomic variations can provide a clear understanding of selection scenarios and speed up the breeding for this important trait in sweetpotato.

## Data availability

The data underlying this study is available from International Potato Center (CIP) Dataverse.

CIP Dataverse: Dataset 1. Dataset for: Continuous Storage Root Formation and Bulking in Sweetpotato http://dx.doi.org/10.21223/P3/IC6ZEY ( Bararyenya *et al*., 2018b)

License: Attribution 4.0 International (CC BY 4.0)
