# Supplementary material for: Continuous Storage Root Formation and Bulking in Sweetpotato
Source: Gates Open Res. 2020 Jul 9;3:83. Originally published 2019 Feb 11. [Version 4] doi: 10.12688/gatesopenres.12895.4 (PMC7267719; doi:10.12688/gatesopenres.12895.4)
Supplement: Peer review history

# Peer review: Reviewer response for version 3

**DOI:** 10.21956/gatesopenres.14302.r28757

**Published:** 2020-06-02

**Authors:** Alfonso del Rio

**Affiliations:** 1 Department of Horticulture, US Potato Genebank, University of Wisconsin-Madison, Sturgeon Bay, WI, USA

I still find lots of details that obscure the data and the important information of the paper. It looks a little better but not quite completed in a way that can allow readers to capture the science and potential applications expected from this study. Some parts are still difficult to read since they do not connect the data with the narrative and the findings. In some cases are repetitive concepts scattered in diverse places.

 The number of figures and tables are too many, 18 in total. In particular I do not think you need that much information.

 I have checked some parts of the paper and have placed some examples of how the authors can synthesize information. My suggestion is that the authors go again all over the manuscript and work on organizing information in a simpler way. Because of the practical value in sweet potato agriculture and sustainability, this study should be easy to read for large audiences in diverse areas.

 To start please note an example in one part of the abstract below. Everything highlighted in bold provides the information. Words between parenthesis are not needed since you can explain those details in results and discussion. I also added some wording suggestions in italics --- 

 .... **Data analysis was done using residual or restricted maximum likelihood (REML). **(in GenStat 18th Edition). (There were strong linear and quadratic trends over time (P<0.001) indicating a) **Data showed non-linear growth pattern within and between locations, seasons, and genotypes for most traits. Some genotypes displayed early initiation and** (a determinate linear) **increase of bulking, while others showed late initiation** (following a quadratic growth pattern). **Broad sense heritability of CSRFAB *was***(would be) **low *probably* due to large GxE interactions *but higher in*** (, however, it was relatively high (50.5%) compared to other yield related) ***other* traits *probably due to*** (indicating) **high genetic influence and *the effectiveness of the methodology*** (accuracy of the developed method to quantify yield overtime)....

**In Experimental Design:**

 The plants of each of the 130 sweet potato genotype were propagated as clonal forms or segregating seeds?

**In Results and Discussion:**

 In *Growth pattern analysis and phenotypic variability across selected CSRFAB sweetpotato traits ---*

*What do you mean when you said ....For most of the traits, their three-way interactions were significant which means in reality that at least one of the 2-way interactions changes across the third factor*. How is this important in the discussion?

 Also it seems to be a contradiction saying that there was high genetic variability across locations and the need to study stability. Please clarify.

***In Variance component analysis and heritability estimates of selected sweetpotato growth traits in a nonlinear model structure***

 You said the same in two parts of the same paragraph .... "This implies that the scale used to measure the **** *trait is precise and the trait is not influenced much by the environment*. Heritability was relatively high for SRNO (67.2%) and CSRFAB (50.5%). The low residual variance and high heritability observed for *CSRFAB i mply that the CSRFAB trait in this study was not greatly influenced by environment and the scale used to measure the trait is precis* e"

**Genetic effects on yield of CSRFAB genotypes in sweetpotato:**

**I placed below another example on how you can be more straightforward in providing a more clear description of your results** : "...We performed a harvest basis analysis and calculated the corresponding heritability for each trait to understand the dynamics of breeding values across harvest times. We targeted the traits that can affect final yield. Thus, the genotypic variances among seven main traits associated with CSRFAB varied from 0 to 72.46 ( Table 6). Genotypic variance was high for HI at 120 DAP (72.46) and VY at 180 DAP (59.91) ( Table 6). Zero variance was recorded for VY (120 DAP), HI (150 DAP), SEN (180 DAP) and weevils (180 DAP). The zero (or negative) variance could be due to an artifact of the algorithm or competition effects between adjacent plots. These results imply no significant change in VY, HI, SEN and weevil damage within population for the respective growth periods. Since heritability is influenced by genetic variance, it was directly affected at the same traits and harvest periods. (ranging from 0 to 67%)..."

**The paragraphs below are highly speculative and possibly wrong if you assume your comments on artifacts in the algorithm. You need to support this better ---** *The 0% heritability implies that the effect of the traits is moving to fixation, that is, its frequency in the population is close to 100%. If we score the effect of the allele (s) regulating the trait having no heritability in the population, the result would mean in genetic terms, that the allele frequency in the population is 100%, therefore the genetic variance at the loci of these genes is zero, so any variance in the corresponding phenotypic traits cannot be attributed to the non-existent genetic variance..*.and the next paragraph. 

 In: **Identification of storage formation and bulking growth patterns and their characterization:**

**I don't find the comparative data from the classic method ---**

 "We characterized in this study growth patterns associated with CSRFAB in sweetpotato and compared the accuracy of CSRFAB scores and the classic method used to measure growth change overtime (compared yield change overtime)....

 Was this measuring rather **consistently** or **effectively **than **accurately**? I don't see data that supports this assertion. 

*...The scoring method developed in this study accurately measured changes in the CSRFAB trait..."*

Is the work clearly and accurately presented and does it cite the current literature?

Yes

If applicable, is the statistical analysis and its interpretation appropriate?

No

Are all the source data underlying the results available to ensure full reproducibility?

No source data required

Is the study design appropriate and is the work technically sound?

Partly

Are the conclusions drawn adequately supported by the results?

Partly

Are sufficient details of methods and analysis provided to allow replication by others?

No

Reviewer Expertise:

Plant breeding and Genetics and, Conservation Genetics.

I confirm that I have read this submission and believe that I have an appropriate level of expertise to confirm that it is of an acceptable scientific standard.

# Response

**Published:** 2020-06-28

**Authors:** Robert Mwanga

**Affiliations:** International Potato Center (CIP), Uganda

**RESPONSE BY AUTHORS (Signed in By Robert Mwanga) TO REVIEWER (Dr. Alfonso del Rio)**

I still find lots of details that obscure the data and the important information of the paper. It looks a little better but not quite completed in a way that can allow readers to capture the science and potential applications expected from this study. Some parts are still difficult to read since they do not connect the data with the narrative and the findings. In some cases are repetitive concepts scattered in diverse places.

**RESPONSE:** *We have modified the text and removed repetitive concepts in the results section.*

The number of figures and tables are too many, 18 in total. In particular I do not think you need that much information.

**RESPONSE:** *We have removed one table and one figure.*

I have checked some parts of the paper and have placed some examples of how the authors can synthesize information. My suggestion is that the authors go again all over the manuscript and work on organizing information in a simpler way. Because of the practical value in sweetpotato agriculture and sustainability, this study should be easy to read for large audiences in diverse areas.

**RESPONSE:** *We have revised the text to make it simpler to read and understand wherever it was not clear. All the suggested areas to be modified have been revised as recommended.*

To start please note an example in one part of the abstract below. Everything highlighted in bold provides the information. Words between parenthesis are not needed since you can explain those details in results and discussion. I also added some wording suggestions in italics --- 

.... **Data analysis was done using residual or restricted maximum likelihood (REML). **(in GenStat 18th Edition). (There were strong linear and quadratic trends over time (P<0.001) indicating a) **Data showed non-linear growth pattern within and between locations, seasons, and genotypes for most traits. Some genotypes displayed early initiation and** (a determinate linear) **increase of bulking, while others showed late initiation** (following a quadratic growth pattern). **Broad sense heritability of CSRFAB *was***(would be) **low *probably* due to large GxE interactions *but higher in*** (, however, it was relatively high (50.5%) compared to other yield related) ***other* traits *probably due to*** (indicating) **high genetic influence and *the effectiveness of the methodology*** (accuracy of the developed method to quantify yield overtime)....

**RESPONSE:** *Text has been modified as suggested.*

**In Experimental Design:**

The plants of each of the 130 sweet potato genotype were propagated as clonal forms or segregating seeds?

**RESPONSE:** *Each genotype was propagated as a clone (not as a botanical seed).*

**In Results and Discussion:**

In *Growth pattern analysis and phenotypic variability across selected CSRFAB sweetpotato traits ---*

*What do you mean when you said ....For most of the traits, their three-way interactions were significant which means in reality that at least one of the 2-way interactions changes across the third factor*. How is this important in the discussion?

Also it seems to be a contradiction saying that there was high genetic variability across locations and the need to study stability. Please clarify.

**RESPONSE:** *The interaction between genotype, location and season were significant as were most of the three-way interactions implying that you have to optimize your growth curve for the 3 factors together, because they modify each other’s effect - see modified text.*

***In Variance component analysis and heritability estimates of selected sweetpotato growth traits in a nonlinear model structure***

You said the same in two parts of the same paragraph .... "This implies that the scale used to measure the *trait is precise and the trait is not influenced much by the environment* . Heritability was relatively high for SRNO (67.2%) and CSRFAB (50.5%). The low residual variance and high heritability observed for *CSRFAB i mply that the CSRFAB trait in this study was not greatly influenced by environment and the scale used to measure the trait is precis* e"

**RESPONSE:** *This has been corrected in the text.*

**Genetic effects on yield of CSRFAB genotypes in sweetpotato:**

**I placed below another example on how you can be more straightforward in providing a more clear description of your results** : "...We performed a harvest basis analysis and calculated the corresponding heritability for each trait to understand the dynamics of breeding values across harvest times. We targeted the traits that can affect final yield. Thus, the genotypic variances among seven main traits associated with CSRFAB varied from 0 to 72.46 ( Table 6). Genotypic variance was high for HI at 120 DAP (72.46) and VY at 180 DAP (59.91) ( Table 6). Zero variance was recorded for VY (120 DAP), HI (150 DAP), SEN (180 DAP) and weevils (180 DAP). The zero (or negative) variance could be due to an artifact of the algorithm or competition effects between adjacent plots. These results imply no significant change in VY, HI, SEN and weevil damage within population for the respective growth periods. Since heritability is influenced by genetic variance, it was directly affected at the same traits and harvest periods. (ranging from 0 to 67%)..."

**RESPONSE:** *The recommended modification has been followed.*

**The paragraphs below are highly speculative and possibly wrong if you assume your comments on artifacts in the algorithm. You need to support this better ---** *The 0% heritability implies that the effect of the traits is moving to fixation, that is, its frequency in the population is close to 100%. If we score the effect of the allele (s) regulating the trait having no heritability in the population, the result would mean in genetic terms, that the allele frequency in the population is 100%, therefore the genetic variance at the loci of these genes is zero, so any variance in the corresponding phenotypic traits cannot be attributed to the non-existent genetic variance..*.and the next paragraph. 

**RESPONSE:****** *The recommended modification has been incorporated.*

In: **Identification of storage formation and bulking growth patterns and their characterization:**

**I don't find the comparative data from the classic method ---**

"We characterized in this study growth patterns associated with CSRFAB in sweetpotato and compared the accuracy of CSRFAB scores and the classic method used to measure growth change overtime (compared yield change overtime)....

**RESPONSE:**

1. *Please, look at Figure 2 growth pattern for classic method using yield against Figure 3 using DCSRFAB scores for the DCSRFAB genotypes.*
2. *Please, also look at Figure 4 growth pattern for classic method using yield against Figure 5 using CSRFAB scores for CSRAB genotypes.*

Was this measuring rather **consistently** or **effectively **than **accurately**? I don't see data that supports this assertion. 

*...The scoring method developed in this study accurately measured changes in the CSRFAB trait..."*

**RESPONSE:****** *The conclusion has been modified as suggested.*

# Peer review: Reviewer response for version 2

**DOI:** 10.21956/gatesopenres.14205.r28324

**Published:** 2020-01-20

**Authors:** Alfonso del Rio

**Affiliations:** 1 Department of Horticulture, US Potato Genebank, University of Wisconsin-Madison, Sturgeon Bay, WI, USA

This study was aimed to determine how continuous storage root formation and bulking vary over diverse genotypes of sweet potatoes from Uganda. This remarks that some genotypes with good CSRFAB can be identified and have the potential to be used to enhance sweet potato productivity.

 It is evident that identifying sources of good traits is significant to promote advances in breeding and agriculture. From that standpoint this study has merit. However, the reviewer finds this manuscript doesn't have good flow and it is difficult to follow.

 One criticism is that the authors build the discussion including excessive statistics and excessive technical details of the statistical analysis. In many cases it was not clear what the findings were as information and concepts become unclear. I would advise the authors to make it simpler, you have to take into consideration that this paper will reach a broad audience with different backgrounds. This study has practical implications, giving a clear view of the results, discussions and analyses could enhance its outreach and practical application from breeding groups in the region. 

 In summary, this manuscript needs major revision. It needs to simplify the statistical analysis to a point of showing what is truly relevant with respect to the variation in traits assessed to explain CSRFAB and the variation among genotypes. This manuscript is too long. 

 Some additional comments:

- In Introduction: The second paragraph, it seems to me that the authors should clarify better if yield increase was because of the effect of breeding or just an increase of planting areas. If the latter this doesn't help much justifying the addition of new breeding forms.
- In Methods: What is a mega-environment for selection? I assume it was as reference to environmental diversity but the term *mega-environmen*t is a bit misleading in my view.
- Results: It is not new to report that clonal forms can respond differently in different environments. Variation levels across multiple environments is often high in field experiments in any crop. How is this result original? Then, you indicate that there is high variability among genotypes across locations + seasons. Wasn't this expected from a variable set of genotypes?
- When the authors indicate that " *this can be explained by the large population effect combined with sample size", *I would suggest clarification in that sentence since I understand the same number of plants and populations were used in the experiments. How were the effects compared on that basis? 
- The parameters that the authors have used are related to storage root development, it seems that indicating that genotype performance increases overtime and is linearly explained is a logical outcome and fully expected. Storage root formation is related to maturity and developmental stages (=time). Maybe you need to clarify that result and explain better.

Is the work clearly and accurately presented and does it cite the current literature?

Yes

If applicable, is the statistical analysis and its interpretation appropriate?

No

Are all the source data underlying the results available to ensure full reproducibility?

No source data required

Is the study design appropriate and is the work technically sound?

Partly

Are the conclusions drawn adequately supported by the results?

Partly

Are sufficient details of methods and analysis provided to allow replication by others?

No

Reviewer Expertise:

Plant breeding and Genetics and, Conservation Genetics.

I confirm that I have read this submission and believe that I have an appropriate level of expertise to confirm that it is of an acceptable scientific standard, however I have significant reservations, as outlined above.

# Peer review: Reviewer response for version 1

**DOI:** 10.21956/gatesopenres.13991.r26950

**Published:** 2019-02-20

**Authors:** Hussein Shimelis

**Affiliations:** 1 African Centre for Crop Improvement, School of Agricultural, Earth and Environmental Sciences , College of Agriculture, Engineering and Science, University of KwaZulu-Natal (UKZN), Pietermaritzburg, South Africa

I suggest the following points for possible consideration:

**Title:**

- It can be adjusted to read: “Variation for Continuous Storage Root Formation and Bulking in Sweetpotato”.

  

**Abstract:**

- Some of the acronyms must be written in full during the first mention e.g. CSRFAB, NaCRRI etc.
- The conclusion is not clear, e.g., which genotypes were selected and recommended for continuous storage root formation and bulking and what yield advantage?

**Introduction:**

- The authors have provided long and detailed information on the overall productivity issues of sweetpotato. Instead, the authors should refocus on the challenges and opportunities of piecemeal harvesting under field conditions, and data and literature on continuous storage root formation and bulking. Problems such as termites, terminal drought stress, multiple and intercropping systems, and limited agricultural lands etc. can be pressing issues for piecemeal harvesting.

 

**Materials and methods:**

- Provide information on the maturity period (in months) of the test genotypes in Table 2. This will influence genotype comparison and recommendation for piecemeal harvesting.
- The 1 to 9 senescence scale should be described in detail, e.g. what is 1, 2, 3, 4, 6, 6, 7, 8 and 9.

 

**Results:**

- In all the Figures it will be interesting if the final yield/score (cumulative yield) of test genotypes are included, not only at 90, 120, 150 and 180 DAP.
- Is there a control harvest (e.g. normal harvest/once off harvest of a given genotype) to compare with cumulative yields over 90, 120, 150 and 180 DAP?

Is the work clearly and accurately presented and does it cite the current literature?

Partly

If applicable, is the statistical analysis and its interpretation appropriate?

Yes

Are all the source data underlying the results available to ensure full reproducibility?

Yes

Is the study design appropriate and is the work technically sound?

Yes

Are the conclusions drawn adequately supported by the results?

Partly

Are sufficient details of methods and analysis provided to allow replication by others?

Partly

Reviewer Expertise:

Plant Breeding, Plant Genetics, Crop Improvement, Quantitative Genetics

I confirm that I have read this submission and believe that I have an appropriate level of expertise to confirm that it is of an acceptable scientific standard.

# Peer review: Reviewer response for version 1

**DOI:** 10.21956/gatesopenres.13991.r26921

**Published:** 2019-02-15

**Authors:** Arthur Villordon

**Affiliations:** 1 LSU Agcenter Sweet Potato Research Station, Chase, LA, USA

**Introduction:**

 This work addresses the subject of what the authors have termed “continuous storage root formation and bulking” in sweetpotato. The stated objective of this study was to investigate the genetic variability of “continuous storage root formation and bulking” and characterize growth patterns at different development stages to identify possible “continuous storage root formation and bulking” sweetpotato genotypes in a Ugandan germplasm collection for possible use as parents in trait improvement.

**Merits:**

 The work addresses an important issue of relatively low sweetpotato storage root yields in Sub Saharan Africa where the crop is considered an important component of food security. Therefore, any work that seeks to understand the biological and environmental constraints of storage root yield has merit for advancing the fundamental understanding of the problem and development of solutions to improve productivity.

**Critique:**

 Although the premise of the study was reasonable, the manuscript has a number of general and specific concerns, chief among this is the apparent oversight of previous work on the subject of storage root formation. There is general scientific consensus of the definition of “storage root formation” within the context of storage root developmental stages. It is unfortunate that the manuscript failed to acknowledge this scientific consensus and how it could have informed the current work and led to the advancement of the understanding of storage root formation in general, and within the context of the study location in particular. A short list of such work is provided in the references section below. I do encourage the authors to review some or all of the listed references, particularly the work of Lowe and Wilson (1974 ^1^). There are many similarities and parallels between the current work and Lowe and Wilson’s methodology and approach. I do believe there is valuable data to be published from this research, but as it stands, I am recommending revision for the manuscript, as I believe that conceptual and structural changes need to be made on the manuscript. I hope that the following critiques will aid the authors in the refining of this manuscript to allow for revision in the future.

 There has been prior work looking at abiotic and biotic constraints on early storage root development (between 30 and 50 days) wherein storage roots were anatomically examined to confirm storage root formation and then storage root number was used to measure the response (please see Solis *et al.* (2014 ^2^) and Gajanayake *et al.* (2014 ^3^) below). A common thread in these prior reports is the confirmation of the anatomical markers of storage root formation or lignification (generally lignified adventitious roots typically do not undergo storage root formation). These anatomical observations are relatively quick and require very basic tools and equipment. In contrast, the present work used visual assessments to indicate “storage root formation” or lignification (third paragraph and figure on page 6). For example, a score of 1 is based on visual observations of “no storage root initiation and no bulking.” Unfortunately, as prior work will show, non-swollen or non-thickened adventitious roots cannot be visually classified as undergoing storage root formation or lignification without anatomical confirmation. As it stands, any analyses or models based on this scale is based on the assumption that a score of 1 or 2=”no storage root initiation”, which may not be accurate. Lowe and Wilson (1974 ^1^) used the same approach as the current work and used an index to quantify thickened storage roots. They made very clear assumptions about their rating scale, in part as a result of their detailed anatomical work on storage root formation, as detailed in another study by Wilson and Lowe (1973 ^4^). The authors are encouraged to review Wilson and Lowe (1974 ^1^) and consider revising along these lines. I encourage the authors to reconsider using the “CSRFAB index” and instead use the quantitative measurements that they are already collected to describe the variation in storage root development and yield among the genotypes.

 If there is one single problem that stands out, one that I find very unfortunate, is this statement found on the 2nd paragraph on page 4: “no study attempted to understand the storage root formation and bulking patterns overtime under field conditions.” Lowe and Wilson (1974 ^1^), referenced above and listed below, conducted such a study, not very different in its conceptual approach from the current work. Lowe and Wilson grew six sweetpotato cultivars in field plots, and subsequently sampled and assessed storage formation and storage root development at weekly intervals during the first 8 weeks of growth, and then at monthly intervals after 8 weeks, for up to 24 weeks. Lowe and Wilson were very keen to recognize that storage root formation can only be determined anatomically, so they developed an index to quantify storage root development based on thickness of roots.

 Finally, I looked up Yanfu *et al.* (1989) and was unable to find the data about storage root initiation occurring as early as 7 days. At this stage, adventitious roots are typically still undergoing primary growth, even in high yielding cultivars. Please cite cultivar or genotype or primary source, if available. In addition, I am unable to locate the data on storage root number ranging from 30 to 112 depending on genotype. Please verify this citation. Perhaps this refers to 30 to 112 g/plant (average root yield)?

**Discussion:**

 The research on which this manuscript is based has generated valuable data on the variability of storage root yields of the sweepotato genotypes in the collection and once the recommended structural and conceptual changes are made, the authors are encouraged to revise. Lowe and Wilson’s (1974 ^1^) work was titled “Comparative Analysis of Tuber Development in Six Sweet Potato (Ipomoea batatas [L.] Lam) Cultivars.” Other than the unfortunate use of “tuber” in this case, perhaps the authors will take a cue from this very relevant prior work. Given that the subject of phenotypic variability of storage root development is under-researched in this geographic area, these findings will have undoubtedly have fundamental and applied merit.

 However, if the authors insist on applying the term “storage root formation” in the context of their work, then they must implicitly define the contextual usage and exceptions relative to what the scientific community has adopted as the generally accepted conceptual and functional definition (please see a sampling of references below). In particular, they need to make the needed assumption that all non-swollen adventitious roots (Score 1) and not all slightly swelling adventitious roots (Score 2) are not undergoing storage root initiation and that anatomical examinations were not conducted. However, I strongly urge the authors to avoid this route as it does not advance our understanding of sweetpotato storage root formation and unnecessarily deviates from the scientific consensus.

Is the work clearly and accurately presented and does it cite the current literature?

Partly

If applicable, is the statistical analysis and its interpretation appropriate?

Partly

Are all the source data underlying the results available to ensure full reproducibility?

Partly

Is the study design appropriate and is the work technically sound?

Partly

Are the conclusions drawn adequately supported by the results?

Partly

Are sufficient details of methods and analysis provided to allow replication by others?

No

Reviewer Expertise:

Relationship between sweetpotato root architecture and storage root development.

I confirm that I have read this submission and believe that I have an appropriate level of expertise to state that I do not consider it to be of an acceptable scientific standard, for reasons outlined above.

## References

[1] : Comparative Analysis of Tuber Development in Six Sweet Potato (Ipomoea batatas (L.) Lam) Cultivars. *Annals of Botany*.1974;38(2) : 10.1093/oxfordjournals.aob.a084814 307-317 10.1093/oxfordjournals.aob.a084814

[2] : Effect of Drought on Storage Root Development and Gene Expression Profile of Sweetpotato under Greenhouse and Field Conditions. *Journal of the American Society for Horticultural Science*.2014;139(3) : 10.21273/JASHS.139.3.317 317-324 10.21273/JASHS.139.3.317

[3] : Quantifying Storage Root Initiation, Growth, and Developmental Responses of Sweetpotato to Early Season Temperature. *Agronomy Journal*.2014;106(5) : 10.2134/agronj14.0067 10.2134/agronj14.0067

[4] : The Anatomy of the Root System in West Indian Sweet Potato (Ipomoea Batatas (L.) Lam.) Cultivars. *Annals of Botany*.1973;37(3) : 10.1093/oxfordjournals.aob.a084729 633-643 10.1093/oxfordjournals.aob.a084729

[5] : The origin and structure of adventitious roots in sweet potato (Ipomoea batatas). *Australian Journal of Botany*.2004;52(4) : 10.1071/BT03152 10.1071/BT03152

[6] : Botany and Physiology: Storage Root Formation and Development.2009; 10.1007/978-1-4020-9475-0_3 13-26 10.1007/978-1-4020-9475-0_3

[7] : A Study of Tuberous Root Initiation in Sweetpotato. *Bulletin of National Agricultural Experimental Station*.1950;68:1-96
